# A Case of Menorrhagia and Treatment

**Published:** 1857-09

**Authors:** E. C. Moyer

**Affiliations:** Talbotton, Geo.


### ARTICLE III.

*A Case of Menorrhagia and Treatment.* By E. C. Moyer, M. D., of Talbotton, Geo.

Mrs. S—, aged 43 years; nervo-bilious temperament; the mother of two children, who died in infancy, consulted me in August, 1855, in reference to her alarming condition. She was suffering from great exhaustion, from excessive Menorrhagic discharge. I found her in the following condition: Excessive nausea, pain in the head, cold extremities, loaded tongue, constipated bowels, pulse 130 per minute, accompanied with disgust for all kinds of aliment, and an inability to retain anything taken per oris. The bowels were opened by enemeta. I ordered Opium, grs. iij; Acetate Plumbi, grs. xij; Calomel, grs. vj, divided into six pills, one to be given every two hours; mustard plaster to the epi-gastrium. The use of the pills increased the already deathly nausea to such an extent as to require their discontinuance; ordered acid sulph. Aromatic, 20 drops, in a half wine glass sweetened water, every two hours. The stomach, after tolerating a few doses, rejected it, though given in combination with infusion of rose leaves, and in every form that it could be administered. Although the frequent application of mustard plasters and turpentine poultices were made, and finally a large blister to the epigastrium, it in no degree lessened the nausea. I then ordered the following mixture: Saturated Tr. Opii, ℥i; Acid Nitric pure, ʒi; Fiat Mist, 10 drops in half wine glass of the infusion of roses, every five hours; the nausea became instantly relieved, and the hemorrhage rapidly declined, and Mrs. S— soon regained her wonted health and vigor.

On the 13th day of August, one year from the menorrhagic attack just referred to, I was called in great haste to see Mrs. S—, who, on my arrival, informed me that she had been flooding sixteen days, not profusely, until within the last few hours. I found the menorrhagic discharge to be very great. She was exceedingly pale, extremities cold, pulse 140 per minute, bowels constipated, tongue heavily coated, and dreadful nausea. I gave ten grs. Blue Mass, followed in four hours with an active dose of Sulphate of Magnesia, acidulated with Sulph, Acid, and its action promoted by enemas of cold water, which in some slight degree relieved the nausea, which, however, soon returned in all its former severity and urgency. A large blister to the epigastrium afforded no relief from her suffering; the Nitric Acid and Tr. Opii were given as in a former attack, Aromatic Sulph. Acid, Acetate Plumbi and Opii, Ergot in Tr. in powder and in infusion, alum and rose water, pulv. Kino Alum, Tannin and Opium, each in their turn were given, without the least relief to the nausea, or in arresting the menorrhagic discharge. Injections per vaginum and per annum with ice cold water were made every two or three hours. Injections per vaginum of Alum water, sol. Acetate Lead, Tanic Acid and Kreasote, Acetate of Zinc, sulphate of Zinc, tincture of the Sesquichloride of Iron, 2 drms. to 6 oz. water, each in their turn, were thoroughly used without in the least checking the hemorrhage. At this stage of the disease and sufferings of my patient, I found her greatly exhausted, almost ex-sanguine, unable to retain anything taken per oris, except dram doses of McMinn's Elixir of Opium. On the 9th day of my attendance, and 25th day of the hemorrhage, I found the womb to be greatly enlarged, *four times its natural size.* A vaginal examination with the speculum uteri, revealed an ulcer, involving the entire ostince, the size of an half dollar, bleeding profusely. I applied a stick of the Nitrate of Silver to the whole ulcerated surface, which only partially arrested the hemorrhage for a few hours. I cauterized the ulcer several times with a solution of the Nitrate of Silver, 10 grs. to the ounce, with no better effect. I applied the Acid Nitrate of Mercury for several days; the ulcer did not improve in the least under its use. Hemorrhage continued unabated; Tannin and Kreasote, the Sulphate and Acetate of Zinc were each in their turn used, with but little if any benefit. I then used tinct. Ferri, Chloridi, drops ij to 4 oz. water, and the hemorrhage was instantly arrested as if by magic. The ulcer soon assumed a healthy appearance, and under the daily use of the black wash, rapidly healed. The nausea disappeared, together with disgust for food. A generous diet was prescribed, and the following Tonic Aperient: ℞—Infusion of Gentian, ℥viij; Infusion Rose Leaves, ℥viij; Sulphate Magnesia, ℥j; Sulphate Ferri, grs. xxx; Acid Sulph. Dilute, ʒij; Fiat. Mist, wine glass full, three times daily. Occasionally a pill of Blue Mass, Ext. Hyosciam and Lupuline was given, in order to keep up the action on the liver. Under the above treatment Mrs. S— rapidly recovered her usual health, strength and spirits, having been confined to her bed fifty days, requiring nineteentwentieths of the time the constant attention of her physician.

On the 2d day of last March, seven months from the last attack, I was again summoned in haste to see Mrs. S—. On my arrival, I found her suffering from a recurrence of another more severe attack of Menorrhagia, all the former symptoms greatly aggravated, with the addition of a pain in the right hypochondrium and right shoulder, unable to lie on the left side, suffering from dreadful nausea and fear of immediate dissolution; liver greatly engorged and torpid, spleen much enlarged. An examination per vaginum, with the speculum uteri, revealed a perfectly sound uteri, and a profuse hemorrhagic discharge from the uterine cavity; the womb was slightly enlarged and anteverted. In order to arouse the liver to action, and as far as possible to disengorge it, I gave ten grains of calomel with Ext. Hyosciamus and Lupulin, followed in four hours with a full dose of Sulphate of Magnesia, acidulated with a few drops of Sulphuric Acid, the operation of the bowels being aided by repeated enemas of cold water. After the bowels were fully unloaded, I gave 60 drops of McMinn's Elixer of Opium, the only form of opium which the stomach would or could tolerate; indeed, the stomach would instantly eject every kind of aliment and medicine. I, as in the previous attack, applied mustard plasters, turpentine poultices, and a large blister to the epigastrium, with no relief to the constant, distressing and urgent nausea, in consequence of which, I was under the necessity of abandoning the administration of every remedial agent per oris, except an occasional dose of McMinn's Elixer of Opium. I used injections of ice cold water every three or four hours per anum; I kept the vaginal canal constantly packed tight, with my favorite Tampon (tow) either dry or saturated with one or the other of the following astringent lotions: Acetate Plumbi, Acetate or Sulphate of Zinc, Sulphate of Alum; Tannin and Kreasote and the tinct. Ferri Chloride, 4 drms. to 8 oz. water. I also kept the uterine orifice constantly plugged with tow. Her hips were elevated, room cool and dark, and the most perfect quietude enjoined; yet, with all the above precautions, day after day, and night after night passed, each day and night finding my patient no better, but gradually growing worse, until the sixteenth day of my constant bedside attendance was reached, my patient no better, but infinitely growing worse, in the most imminent danger, prostrated from irritation, and the long and unchecked hemorrhagic discharge, almost articulo mortis. In this alarming state of things what was I to do? Every effort heretofore had been unavailing, perfectly abortive. In this extremity, I felt that I had one more resource left. I resolved to try it, although its use might be attended with fatal consequences, yet I felt that the end justified the means, viz: to inject the cavity of the womb with the Nitrate of Silver. Having no suitable instrument, I passed a male silver catheter through four inches of a No. 10 gum elastic catheter, leaving two inches above the end of the silver catheter, into which I inserted the point of a two inch glass syringe, charged with a solution of the Nitrate of Silver, 10 grs. to the ounce of water. I injected with much force into the uterine cavity, its contents; and to my inexpressible delight, as by magic, the hemorrhage was instantly arrested. The injection caused much nausea and pain, which was soon relieved by 100 drops McMunn's Elixir of Opium. There was a slight return of the hemorrhage, the next day. I repeated the injection; Mrs. S. experienced but trifling pain, no nausea; the hemorrhage ceased, together with the nausea, neither afterwards returned. I continued the mercurial preparation with ext. Hyosciamus and Lupuline every third or fourth night, followed in the morning with a good dose of Sulphate Magnesia, acidulated with Sulphuric Acid. In conjunction with the following Tonic Aperient, ℞ Infusion Genitian, ℥viiss; Infusion Roses, ℥viiss; Syrup Rhacadas, ℥i; Sulphas Ferri grains, Magnesia Sulphas, ℥i; Acid Sulph. Dilute, ʒii; Fiat Mist, Dose, a wine glass full, three times a day, aided by a generous diet. Mrs. S. Rapidly regained her former health and vivacity. And although she has menstruated regularly, she has suffered no return of the hemorrhagic symptoms, and to-day, 12th August, 1857, she is in the enjoyment of better health and spirits, than for the last 25 years.

I regard the hemorrhagic attacks, in the cases referred to, as purely cases of passive Menorrhagia, modified by the condition of the stomach, liver, and uterus. The stomach in an highly irritable condition, (sympathetically affected,) the liver torpid and greatly engorged, and the womb slighly enlarged and anteverted. Indeed, I conceive that the large majority of the cases of menorrhagia are of the passive kind; if not so primarily, they become so secondarily, from the force of circumstances. In addition to the above plan of treatment, I would suggest the free use of acidulated drinks, mustard plaster, or blister between the shoulders, cold hip bath, where the patient is able to take it, or the cold spunging the entire body every morning, and after wiping the person entirely dry, friction to be freely made with a salt towel, and a most generous diet. In concluding this report, I must enter my protest against the too free use of astringents, per vaginum; in most cases I believe them worse than useless; from long experience, especially in the case of Mrs. S., I am convinced that astringent lotions injected into the vaginal cavity create an irritation in the neck of the womb and vagina, and act injuriously upon the principle of "ubi irritatio ibi fluxus."

